# Research Progress on the Effect of *Bacillus velezensis* on Disease Resistance of Aquatic Animals

**DOI:** 10.3390/cimb48080755

**Published:** 2026-07-25

**Authors:** Xue Yan, Tingyu Zhu, Mengting Xu, Yize Wang, Shuxin Zhao, Mingxu Xie, Chenglong Wu

**Affiliations:** School of Life Science, Huzhou Normal University, 759 East 2 nd Road, Huzhou 313000, China

**Keywords:** *Bacillus velezensis*, aquatic animals, disease resistance, mechanisms

## Abstract

With the rapid expansion of intensive aquaculture, the frequent outbreak of diseases and the overuse of antibiotics have become increasingly critical issues. Therefore, developing green and efficient antibiotic alternatives is essential for the sustainable growth of the industry. As a novel probiotic, *Bacillus velezensis* has shown great potential in improving the disease resistance of aquatic animals, owing to its excellent spore-forming ability, broad-spectrum antibacterial activity, and immunoregulatory functions. This paper systematically reviews the main mechanisms by which *B. velezensis* enhances disease resistance in aquatic animals, including directly inhibiting pathogens through the secretion of various lipopeptides and polyketides such as surfactin, iturin, and fengycin; interfering with pathogen quorum sensing systems via quorum quenching enzymes, thereby suppressing virulence factor expression and biofilm formation; modulating intestinal microbiota structure to increase the abundance of beneficial bacteria while reducing pathogenic proliferation; and activating the non-specific immune system of the host to upregulate immune-related indicators such as acid phosphatase, superoxide dismutase, and key cytokines (such as *IL-1β*, *TNF-α*). In addition, this review summarizes the application effects of *B. velezensis* on improving aquaculture water quality and promoting host growth. Furthermore, the potential ecological impacts on natural microbial communities and strain-dependent biosafety risks are evaluated. Finally, current research limitations—such as strain-specific functional differences, insufficient in-depth analysis of mechanisms, and inconsistent application outcomes—are discussed. Future prospects for promoting the efficient and stable application of *B. velezensis* in aquaculture through strategies including gene editing, multi-omics integration, and precise formulation compounding are also proposed.

## 1. Introduction

With the development of the aquaculture industry toward an intensive and high-density mode, the deterioration of aquaculture environment and the frequent occurrence of diseases have become major bottlenecks limiting the sustainable development of the industry [1,2]. According to the Food and Agriculture Organization of the United Nations, diseases cause billions of dollars in annual economic losses to global aquaculture [3,4]. For an extended period, the widespread or even excessive use of antibiotics has been the primary strategy for disease prevention and control. However, this practice not only increases pathogenic bacterial resistance and drug residues but also poses potential risks to environmental ecology and human health [5,6]. Consequently, the search for safe, efficient, and eco-friendly antibiotic substitutes has become a research priority in the global aquaculture sector.

Probiotics are widely regarded as one of the most promising alternatives to antibiotics, owing to their multifaceted benefits including promoting host growth, enhancing immunity, inhibiting pathogens, and improving water quality [7,8,9]. Among various probiotics, *Bacillus* spp. have become the preferred feed additives due to their ability to form highly stress-resistant spores, which confer excellent storage stability and gastrointestinal survival [10,11,12]. Within this genus, *Bacillus velezensis* has garnered substantial attention in recent years as a newly recognized member [13,14]. Initially misclassified as a synonym of *Bacillus amyloliquefaciens*, its status as a distinct species was confirmed in 2016 through advances in genomic research [15]. *B. velezensis* is widely distributed in soil, aquatic environments, animals, and plants. It secretes various digestive enzymes (e.g., protease, amylase, lipase) and abundant secondary metabolites (e.g., lipopeptides, polyketides), and exhibits strong inhibitory effects against a broad range of aquatic pathogens [16].

Therefore, this paper systematically reviews recent research progress on the role of *B. velezensis* in enhancing disease resistance in aquatic animals, with an emphasis on its underlying mechanisms. It also identifies current research limitations and proposes future directions, aiming to provide a theoretical basis for the further development and scientific application of this bacterium in aquaculture.

## 2. Overview of the Probiotic Properties of *B. velezensis*

### 2.1. Biological and Physicochemical Characteristics

*B. velezensis* belongs to the genus *Bacillus* and is an aerobic, Gram-positive bacterium. The cells are short rod-shaped, often arranged in pairs, typically measuring (0.5–0.7) μm × (2.0–3.0) μm, and are motile by peritrichous flagella [17]. On solid culture media, its colonies are usually circular, flat, with irregular edges, moist and shiny surfaces, wrinkled, and exhibit an off-white or opaque color with a viscous texture [18,19]. This bacterium is capable of forming endogenous spores, which are oval in shape and possess strong stress resistance, enabling tolerance to extreme environments such as high temperature, desiccation, acid/base conditions, and electromagnetic radiation [20].

In terms of physiological and biochemical characteristics, *B. velezensis* shows positive catalase, a positive Voges–Proskauer (V-P) test and positive methyl red (M-R) test. It can reduce nitrate, hydrolyze starch and gelatin, and utilize a variety of sugars such as glucose, sucrose, mannose, cellobiose and arabinose, but cannot utilize rhamnose, xylose and raffinose [21,22]. The optimum growth temperature for this bacterium ranges from 25 to 37 °C, with slow growth below 20 °C. The optimum pH range is 5.0–9.0, while growth is markedly reduced at pH values below 3.0 or above 11.0. Additionally, it can grow at salt concentrations of 5–60% [23]. Regarding gastrointestinal tolerance, *B. velezensis* shows moderate tolerance to bile salts and acidic conditions, surviving at bile salt concentrations of 0.3–0.4% and at pH 2.0–4.0 in simulated gastric fluids [18]. Although the survival rates at pH 2.0, 3.0, and 4.0 were 12.77% and 14.65%, respectively—values that are not exceedingly high—the same study demonstrated that cells could resume proliferation upon transfer from acidic gastric conditions (pH 3.0 or 4.0) to a neutral intestinal environment (pH 6.8). This recovery capacity supports its potential for gastrointestinal colonization and probiotic efficacy [18]. In terms of safety, *B. velezensis* generally displays γ-hemolysis and is sensitive to various antibiotics (e.g., tetracycline, erythromycin, chloramphenicol, ciprofloxacin). It does not carry transferable antibiotic resistance genes or major enterotoxin genes, and is therefore considered to have good biosafety for aquatic animals and livestock [24,25].

For enzyme production characteristics, *B. velezensis* possesses a powerful enzyme secretion system capable of producing various extracellular hydrolases. Both genomic analysis and phenotypic identification have confirmed that *B. velezensis* can secrete multiple digestive enzymes, including proteases, amylases, lipases, and cellulases [26]. Studies have shown that the *B. velezensis* HF-14,109 strain, isolated from the intestine of healthy common carp (*Cyprinus carpio* L.), carries dozens of glycoside hydrolase genes and efficiently expresses amylases, proteases, lipases, and β-glucanases [27]. These enzymes not only directly assist aquatic animals in breaking down polysaccharides and large molecular proteins in feed but also degrade anti-nutritional factors in plant-based protein sources, thereby improving feed conversion rates and enhancing the general health and basal disease resistance of aquatic animals [27]. Similarly, Wang et al. (2024) reported that the *B. velezensis* D6 strain, isolated from fermented food, exhibited coordinated co-expression of α-amylase and protease during fermentation, with the transcriptional levels of both enzymes showing a synchronous upward trend within 0–24 h [28]. This synergistic enzyme production mechanism enables efficient hydrolysis of starchy substrates such as corn and rice, as well as large molecular proteins in soybean meal, suggesting that *B. velezensis* strains from different sources possess the metabolic potential to degrade complex substrates through multi-enzyme synergy.

In addition to conventional digestive enzymes, *B. velezensis* can secrete enzymes with specialized degradation functions, such as laccases, cytochromes P450, and specific carboxylesterases [29,30]. These specialized enzymes can synergistically cleave polyethylene derivatives and hydrolyze toxic plasticizers (e.g., phthalates), effectively reducing the toxic stress caused by these substances on the hepatopancreas of aquatic animals [29,30]. Furthermore, the proteases and cellulases secreted by *B. velezensis* can rapidly degrade large molecular organic matter such as residual feed and feces deposited on the pond bottom, preventing substrate deterioration. They can also degrade or disrupt biofilms formed by opportunistic aquatic pathogens (e.g., *Vibrio* spp., *Aeromonas* spp.), depriving pathogens of their ecological niches and thereby reducing the risk of disease outbreaks at the environmental source [31,32]. These enzymes play multiple roles in the probiotic process, promoting nutrient digestion and absorption, improving intestinal health, and creating favorable physiological conditions for the host to resist pathogenic infections.

### 2.2. Genomic Characteristics

The genome size of *B. velezensis* is approximately 3.9 Mb, with a GC content of about 46% [33]. The most prominent feature of its genome is the presence of numerous gene clusters related to the biosynthesis of secondary metabolites [13]. These gene clusters encode non-ribosomal peptide synthetases (NRPSs) and polyketide synthases (PKSs), which are responsible for producing a wide variety of antimicrobial substances [34]. For instance, complete gene clusters encoding surfactin, iturin, fengycin, bacilysin, macrolactin, and difficidin have been identified from the genomes of different strains, and the total length of these clusters can account for more than 8% of the entire genome [35]. This rich genetic endowment explains, at the genomic level, why *B. velezensis* has become a broad-spectrum and highly effective biocontrol agent, providing a solid molecular foundation for its use as an antibiotic alternative in aquaculture. Regarding safety, no hemolysin or virulence factor genes have been detected in the genome of *B. velezensis*, and most strains are sensitive to antibiotics, making them suitable for use as feed additives [36]. Furthermore, genomic differences exist among strains from different sources; strains of aquatic origin exhibit higher similarity to each other, suggesting a certain degree of host adaptation [34,35].

## 3. Effects of *B. velezensis* on Disease Resistance in Aquatic Animals

At the antimicrobial level, studies have shown that *B. velezensis* strains isolated from the intestines of aquatic animals exhibit broad-spectrum inhibitory activity against various Gram-negative pathogens, including *Aeromonas hydrophila*, *Aeromonas veronii*, and *Vibrio parahaemolyticus* [23,37,38,39]. At the immune activation level, *B. velezensis* significantly increases the survival rate of aquatic animals after pathogen challenge, as demonstrated in European sea bass (*Dicentrarchus labrax*) [40], crucian carp (*Carassius auratus*) [41], grass carp (*Ctenopharyngodon idella*) [42], Nile tilapia (*Oreochromis niloticus*) [43], oriental river prawn (*Macrobrachium nipponense*) [44], and Pacific white shrimp (*Litopenaeus vannamei*) [45]. Notably, a study by Zhang et al. (2019) confirmed that dietary supplementation with *B. velezensis* strains C-11, S-22, L-17, and S-14 maintained the integrity of intestinal villus structure and upregulated the expression of immune-related genes in crucian carp, indicating that intestinal morphological protection and immune activation serve as structural and functional bases for enhanced disease resistance [46]. Furthermore, in vitro co-culture experiments have shown that *B. velezensis* reduces the hemolytic activity and virulence of *A. hydrophila*, providing evidence from the perspective of pathogen regulation to support its disease resistance mechanism [42]. Collectively, these findings indicate that *B. velezensis* can improve the survival rate of pathogen-infected aquatic animals and enhance their ability to resist pathogenic infections. To provide a systematic overview of the strain-specific probiotic effects described above, the key findings from representative *B. velezensis* strains across different aquatic animal species are summarized in Table 1.

Although the studies summarized above demonstrate the remarkable probiotic efficacy of *B. velezensis*, direct comparative studies with other commonly used probiotic species remain scarce. Available evidence suggests that *B. velezensis* may confer superior or comparable benefits relative to other *Bacillus* species in certain aspects. A comparative study on *Penaeus vannamei* revealed that dietary supplementation with *B. velezensis* 26.3 and *Bacillus subtilis* BSXE-1601 both significantly improved growth performance, but *B. velezensis* induced significantly higher serum acid phosphatase, alkaline phosphatase, and superoxide dismutase activities than *B. subtilis*, indicating a stronger immunostimulatory effect [62]. Both species outperformed *Bacillus cereus* BCXE-01 in eliciting immune responses, while all three *Bacillus* species significantly enhanced resistance against *Vibrio* parahaemolyticus challenge [62]. A comprehensive literature survey further indicates that *B. subtilis* remains the most extensively studied probiotic in fish farming (n = 185 published studies), followed by *B. licheniformis* (n = 61), *B. amyloliquefaciens* (n = 42), and *B. velezensis* (n = 34), highlighting the relatively recent emergence of *B. velezensis* as a probiotic candidate [63]. This comparative evidence underscores the strain-dependent nature of probiotic efficacy and suggests that *B. velezensis* holds competitive advantages in immune modulation, though further head-to-head studies across multiple host species are warranted to establish comprehensive selection criteria.

## 4. Potential Mechanisms by Which *B. velezensis* Affects Disease Resistance in Aquatic Animals

**Figure 1:**
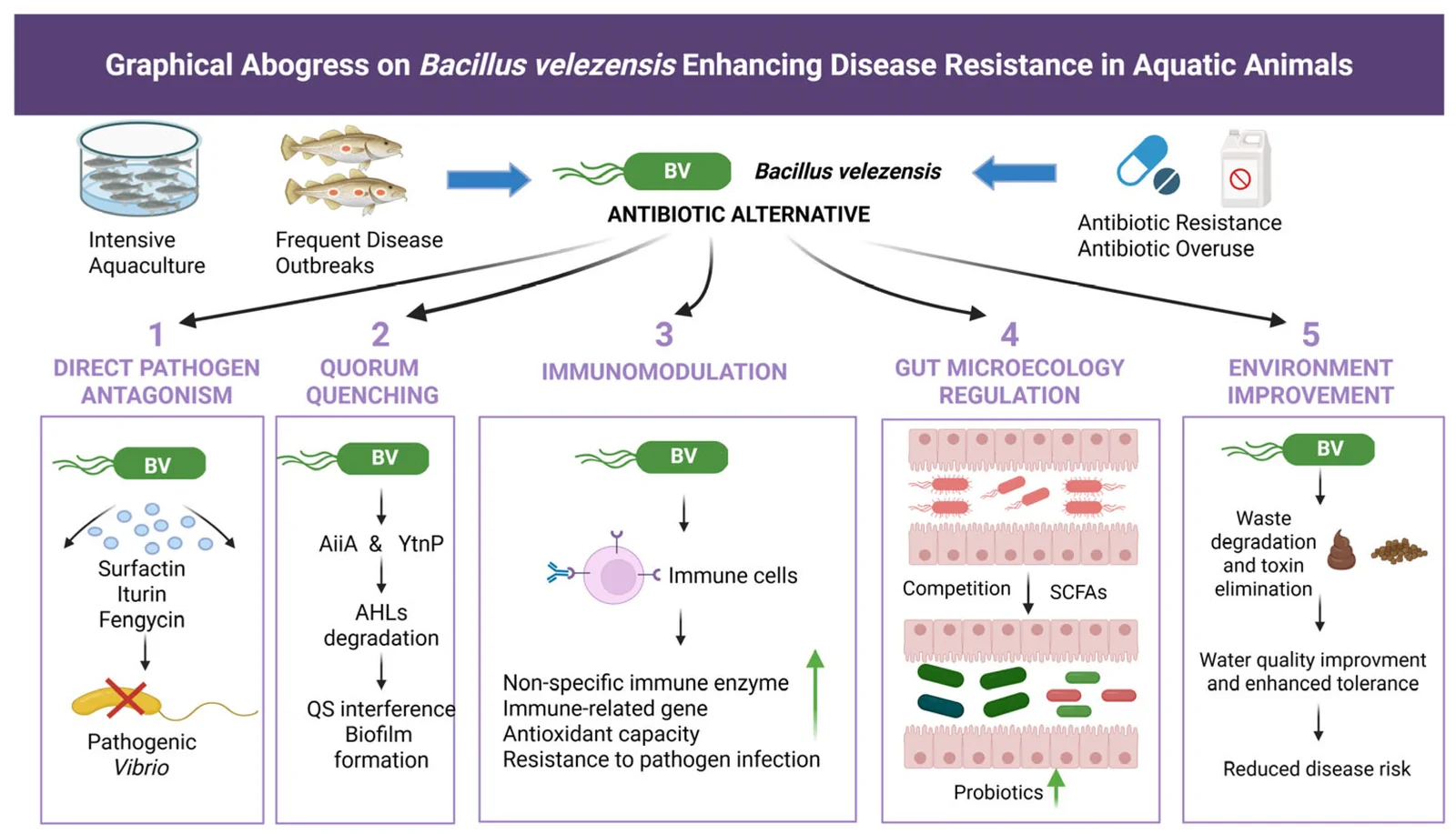
Schematic overview of the mechanisms by which *Bacillus velezensis* (BV) enhances disease resistance in aquatic animals. BV acts through five interconnected pathways: (**1**) direct pathogen inhibition via antimicrobial lipopeptides (surfactin, iturin, fengycin); (**2**) quorum quenching through AHL-degrading enzymes (AiiA, YtnP) that disrupt bacterial communication and biofilm formation; (**3**) host immunomodulation by enhancing non-specific immune enzymes, immune-related gene expression, and antioxidant capacity; (**4**) regulation of intestinal microbiota by promoting beneficial bacteria and suppressing opportunistic pathogens; and (**5**) improvement of water quality through degradation of ammonia nitrogen and organic waste. These synergistic mechanisms collectively reduce disease risk and support sustainable aquaculture. BV, *Bacillus velezensis*; AHLs, acyl-homoserine lactones; QS, quorum sensing; SCFAs, short-chain fatty acids.

Figure 1 provides a comprehensive overview of the major mechanisms through which *B. velezensis* enhances disease resistance in aquatic animals, covering five interconnected pathways: direct antagonism via antimicrobial lipopeptides, quorum quenching through enzymatic degradation of AHLs, immunomodulation of the host immune system, regulation of intestinal microecology, and improvement of the aquaculture environment. These mechanisms function synergistically to reduce pathogen virulence, enhance host immunity, stabilize gut microbiota, and mitigate environmental stressors, ultimately contributing to improved disease resistance and sustainable aquaculture production.

### 4.1. Direct Antagonistic Effects

*B. velezensis* achieves direct inhibition and killing of pathogens by secreting various highly active antimicrobial substances. *B. velezensis* produces a diverse array of antimicrobial agents, including proteins and non-ribosomally synthesized antibiotics. Among the latter are three families of lipopeptides—surfactin, iturin, and fengycin—along with three groups of polyketides, namely macrolactin, bacillaene, and difficidin, as well as other antibiotic compounds such as bacilysin [16]. The genetic basis underlying this antimicrobial capacity has been confirmed in multiple *B. velezensis* strains. For instance, genomic analysis of strain JW revealed multiple gene clusters responsible for bacteriocins, polyketides, and non-ribosomal peptides; feeding trials further demonstrated that this strain enhances survival in fish challenged with *A. hydrophila* and exhibits broad inhibitory activity against various aquatic bacterial pathogens [41]. Similarly, strain CPA1-1 carries 11 genes involved in the production of lipopeptides, polyketones, and antimicrobial proteins, highlighting its strong antibacterial potential for aquaculture disease control [44]. Likewise, strain FS26 harbors five conserved gene clusters (bacilysin, bacillibactin, fengycin, bacillaene, and macrolactin H) that produce antimicrobials effective against bacterial, fungal, and cyanobacterial pathogens in aquaculture [34]. Collectively, these findings underscore the robust and genetically encoded antimicrobial machinery of *B. velezensis*, positioning it as a promising probiotic for disease prevention in aquaculture.

### 4.2. Quorum Quenching Activity

Quorum sensing (QS) is a bacterial communication mechanism through which pathogens coordinate group behaviors, including biofilm formation and virulence factor secretion, in response to the density of signaling molecules [32,64]. Under QS regulation, *Bacillus* species are capable of synthesizing various secondary metabolites, including lipopeptides and polyketones, which exhibit antibacterial, antiviral, and antitumor properties [65,66]. Among the well-characterized quorum quenching (QQ) enzymes widely distributed in *Bacillus* is the acyl-homoserine lactonase AiiA, which specifically recognizes and degrades acyl-homoserine lactone (AHL) signal molecules produced by Gram-negative bacteria. By interfering with biofilm formation and toxin release in AHLs/Lux-mediated pathogens, AiiA effectively disrupts QS signaling [67]. Studies have confirmed that the genome of *B. velezensis* (e.g., strain DH82) harbors genes encoding lactonases such as AiiA and YtnP, which degrade acyl-homoserine lactones (AHLs)—the key signaling molecules of Gram-negative pathogens including *Vibrio parahaemolyticus* [39]. Using *Chromobacterium violaceum* MK as a reporter strain, Monzón-Atienza et al. (2022) demonstrated that *B. velezensis* D-18 exhibits strong QQ activity against *Vibrio anguillarum* 507 by producing lactonase enzymes that degrade both short- and long-chain AHLs, thereby reducing bacterial communication and biofilm formation [68].

### 4.3. Modulation of the Host Immune System

Numerous studies have demonstrated that *B. velezensis*, as a probiotic, can significantly enhance the immune function and disease resistance of aquatic animals through multiple pathways, including increasing non-specific immune enzyme activities, regulating the expression of immune-related genes, improving antioxidant capacity, and enhancing intestinal mucosal immunity.

(1) Enhancement of non-specific immune enzyme activities

Dietary supplementation with *B. velezensis* has been shown to significantly increase the activities of key immune enzymes—such as acid phosphatase (ACP), alkaline phosphatase (AKP), lysozyme, superoxide dismutase (SOD), and catalase (CAT)—in the serum or tissues of various fish species. For instance, in crucian carp, feeding with *B. velezensis* JW significantly elevated serum ACP, AKP, and glutathione peroxidase (GSH-Px) activities [41]. In Atlantic salmon (*Salmo salar*), administration of a probiotic mixture containing *B. velezensis* V4 and *Rhodotorula mucilaginosa* increased serum ACP and immunoglobulin M (IgM) levels, while decreasing nitric oxide (NO), alanine aminotransferase (ALT), and aspartate aminotransferase (AST) levels, suggesting reduced liver damage and inflammatory responses [59,60]. In hybrid grouper (*Epinephelus Lanceolatus* ♂ *× E. Fuscoguttatus* ♀), dietary *B. velezensis* K2 significantly enhanced serum ACP activity [52]. Moreover, strains such as *B. velezensis* FiA2, LH023, GY65, and R-71003 have been reported to increase the activities of lysozyme, SOD, and CAT in crucian carp, grass carp, mandarin fish (*Siniperca chuatsi*), and common carp, respectively [50,51,54,55]. Similarly, in shrimp and prawns, *B. velezensis* significantly elevated the activities of ACP, AKP, SOD, CAT, and lysozyme [44,45,48]. A comprehensive summary of strain-specific immune enzyme enhancements across different host species is provided in Table 1.

(2) Regulation of immune-related gene expression

*B. velezensis* bidirectionally modulates the expression of pro-inflammatory and anti-inflammatory cytokines, while also upregulating antimicrobial peptides and key signaling pathway molecules. For example, in the head kidney of crucian carp, the JW strain significantly upregulated the expression of interferon-gamma (*IFN-γ*) and tumor necrosis factor-alpha (*TNF-α*) [41]. Conversely, in the intestine of grass carp, strain LH023 downregulated pro-inflammatory cytokine genes (*IFN-γ2*, *c-Rel*, *NF-κB*, *p52*, *IKKβ*, *IKKγ*) and concurrently upregulated anti-inflammatory cytokine genes (*S6K1*, *IL-4*, *IL-10*) [54]. Similarly, in hybrid grouper, the K2 strain enhanced the expression of lysozyme, the antimicrobial peptide piscidin, IgM, and the adaptor protein MyD88, while downregulating TLR3 and several inflammatory chemokines [52]. In European sea bass, *B. velezensis* D-18 significantly increased serum bactericidal capacity, nitric oxide levels, and lysozyme activity, alongside upregulating the pro-inflammatory cytokines *il1b*, *tnfa*, *cox2*, and the antimicrobial peptide dicentracin [68]. Likewise, in rainbow trout (*Oncorhynchus mykiss*), combined administration of *B. velezensis* with *Lactobacillus sakei* positively influenced immune-related gene expression [61]. In common carp, *B. velezensis* R-71003 enhanced immunity by activating the TLR4 signaling pathway, thereby regulating the expression of *TLR4*, *MyD88*, *IRAK1*, *TRAF6*, *TRIF*, and *NF-κB* [55]. Furthermore, in zebrafish (*Danio rerio*), strain T23 increased total antioxidant capacity, lysozyme activity, and complement C3 and C4 levels in skin mucus [58]; in addition, strains T1 and T23 indirectly modulated the expression of innate immune-related genes by influencing the gut microbiota of germ-free zebrafish [57]. The immunomodulatory effects of other strains, including FiA2 and LF01, are also summarized in Table 1 [43,50].

(3) Enhancement of antioxidant capacity

*B. velezensis* significantly enhances the antioxidant defense system of aquatic animals. In Atlantic salmon, a multi-strain probiotic formulation combining *B. velezensis* V4 and *Rhodotorula mucilaginosa* markedly increased serum total SOD, GSH, GSH-Px, CAT, and total antioxidant capacity (T-AOC), while concurrently reducing malondialdehyde (MDA) content [60]. Similarly, in common carp, strain R71003 elevated CAT and SOD activities and lowered MDA levels [55]. In mandarin fish, strain GY65 enhanced CAT and SOD activities [51]. In the oriental river prawn, strain CPA1-1 significantly increased SOD activity [44]. In Pacific white shrimp, strain BV007 enhanced respiratory burst and the activities of SOD, CAT, and AKP [48]. Collectively, these findings demonstrate that *B. velezensis* alleviates oxidative stress by scavenging reactive oxygen species and attenuating lipid peroxidation. Additional examples of antioxidant enzyme enhancement (SOD, CAT, GSH-Px) are listed for multiple strains in Table 1.

(4) Enhancement of resistance to pathogen infection

The immunomodulatory effects described above ultimately confer significantly enhanced resistance to pathogenic infections. Across a wide range of aquatic species, dietary supplementation with *B. velezensis* has been shown to improve survival following challenge with various bacterial pathogens. Among fish species, the protective effects are particularly notable. In Atlantic salmon challenged with *Aeromonas salmonicida*, survival increased from 6.7% to 26.7% [60]. In hybrid grouper challenged with *Vibrio harveyi*, survival rose from 25% to 55% [52]. In European sea bass, strain D-18 significantly improved survival compared to the control group [68,69]. Similarly, in crucian carp, grass carp, mandarin fish, common carp, and zebrafish, various *B. velezensis* strains—including FiA2, LH023, GY65, R-71003, T1, and T23—consistently enhanced post-challenge survival or reduced cumulative mortality [50,51,54,55,57]. Notably, among the large yellow croaker-derived strains tested in zebrafish, T23 exhibited greater efficacy than T1 [57]. In crustaceans, similar protective effects have been observed. In oriental river prawn, strain CPA1-1 led to higher survival than the control following pathogen challenge [44]. In Pacific white shrimp, strain BV007 enhanced immune responses against *Vibrio parahaemolyticus* infection [48].

In summary, *B. velezensis* significantly enhances disease resistance in aquatic animals such as fish and shrimp through multi-target, multi-pathway immunomodulatory mechanisms, including increasing non-specific immune enzyme activities, regulating the cytokine network, activating the TLR signaling pathway, enhancing antioxidant capacity, and optimizing intestinal microbiota structure. The survival improvements conferred by various strains against different pathogens, as detailed in Table 1, consistently support these immunomodulatory benefits. Strain-specific differences in immunomodulatory efficacy exist; for example, strain T23 outperforms T1 in growth promotion and immune regulation [57], and combining *B. velezensis* with other probiotics (e.g., *Rhodotorula mucilaginosa*) or functional substances (e.g., chitosan, nano-silica) can produce synergistic effects [60,69]. These findings provide a solid theoretical basis for the development and application of *B. velezensis* as a probiotic in aquaculture.

### 4.4. Regulation of the Host Intestinal Microecology

The homeostasis of the intestinal microbiota is a core factor in maintaining host health [70]. Numerous studies have shown that dietary supplementation with *B. velezensis* can reshape the intestinal microbiota structure of aquatic animals. The typical features include an increase in the abundance of beneficial bacteria and suppression of opportunistic pathogens. For example, in crucian carp, supplementation with *B. velezensis* strain FiA2 significantly increased the relative abundance of Firmicutes and Actinobacteria in the intestine, while the abundance of the potential pathogen *Plesiomonas* was markedly reduced [50]. Functionally, Firmicutes are not only involved in carbohydrate metabolism but also secrete soluble factors and metabolites such as short-chain fatty acids (SCFAs), thereby regulating intestinal epithelial cell function and inducing the production of cytokines and B-cell activating factors [71,72]. Actinobacteria are also a dominant phylum in the healthy gut, playing key roles in maintaining intestinal homeostasis, energy metabolism, and immune regulation [73]. In contrast, *Plesiomonas* has been confirmed to cause infections in various aquatic hosts [74]. Similar beneficial regulatory effects have been verified in other fish species. For instance, adding *B. velezensis* LH023, originally isolated from grass carp intestine, back into the feed also increased the abundance of beneficial gut bacteria while reducing harmful microorganisms [54]. Additionally, *B. velezensis* BV007, isolated from the intestine of Pacific white shrimp, significantly increased the abundance of potential probiotics (*Bacillus* spp.) and decreased the abundance of potential pathogens (*Vibrio* spp.) in the shrimp gut [48]. This effect may be attributed to competition for nutrients and intestinal ecological niches by *B. velezensis*, as well as the direct inhibition of pathogens by its secreted antimicrobial substances.

It is worth noting that the regulation of intestinal microbiota by probiotic strains does not simply involve the elimination of specific bacterial groups; rather, it reestablishes a new homeostatic balance through dynamic ecological shifts. For instance, *B. velezensis* T23 and its nuclease-treated derivative were found to increase the abundance of *Aeromonas* (within Proteobacteria) while simultaneously enhancing the abundance of *Cetobacterium* (within Fusobacteria) [58]. Fusobacteria represent one of the dominant phyla in the fish intestine and can improve host metabolism by producing short-chain fatty acids, vitamin B12, and bacteriocins [75]. *Cetobacterium*, a key genus within this phylum, has been shown to improve liver and intestinal health and enhance antiviral immunity in zebrafish [76,77]. In the T23-treated group described above, the expansion of Fusobacteria may be sufficient to counterbalance the excessive proliferation of Proteobacteria, thereby reshaping the intestinal microbiota and contributing to the maintenance of homeostasis.

Furthermore, the administration strategy of probiotics plays a critical role in shaping the dynamics of microbial diversity. A trial on Nile tilapia revealed that continuous feeding with *B. velezensis* LF01 for nine weeks resulted in no significant difference in intestinal microbiota diversity between the treatment and control groups. In contrast, a significant peak in the diversity index was observed at week six in the group receiving an intermittent feeding strategy. Notably, the levels of *Edwardsiella* and *Plesiomonas*—two important fish pathogens—were markedly lowered in the intestines of the LF01-treated group, suggesting that probiotic supplementation effectively curbs the proliferation of endogenous opportunistic pathogens [43]. These findings are corroborated by studies in rainbow trout, where dietary supplementation with *B. velezensis* remodeled the intestinal microbial structure and increased the relative abundance of various potentially beneficial bacteria, including *Ruminococcus*, Lachnospiraceae UCG-004, *Leptotrichia*, *Bacillus coagulans*, Porphyromonadaceae, anaerobic cocci, and photobacteria [61].

### 4.5. Improvement of the Aquaculture Environment

The application of *B. velezensis* offers considerable environmental benefits. Studies have demonstrated that introducing *B. velezensis* into aquaculture water effectively facilitates the degradation of organic waste and reduces the concentrations of harmful substances, including ammonia nitrogen, nitrite, total nitrogen, and total phosphorus. Regarding nitrogen removal, *B. velezensis* M3-1 exhibits a distinctive synergistic denitrification capacity. The nitrate reduction process mediated by this strain efficiently eliminates residual nitrate produced by anaerobic ammonium oxidation, thereby markedly improving total nitrogen removal efficiency in aquaculture wastewater [78]. Similarly, in pond-cultured channel catfish (*Ictalurus punctatus*), dietary supplementation with *B. velezensis* AP193 significantly enhanced water quality, reducing total phosphorus, total nitrogen, and nitrate concentrations by 19%, 43%, and 75%, respectively [47]. This purification effect is attributable to both the bacterial assimilation of nitrogen and phosphorus and the phytase secreted by AP193, which degrades phytic acid—an anti-nutritional factor present in plant-based feeds—thereby lowering phosphorus excretion in feces and potentially inhibiting pathogen growth indirectly through reduced inositol levels.

Notably, *B. velezensis* LG37 not only degrades ammonia nitrogen and organic matter in aquaculture water with high efficiency but also significantly enhances the tolerance of cultured animals to ammonia nitrogen stress. Supplementation of grass carp feed with LG37 resulted in ammonia nitrogen and feed protein degradation rates of 55.5% and 73.6%, respectively, while the safe tolerance concentration of fish to unionized ammonia increased by 51.63%, demonstrating the dual functions of water purification and ammonia toxicity mitigation [53]. Furthermore, *B. velezensis* CPA1-1 has been shown to degrade ammonia nitrogen and nitrite in the culture water of the oriental river prawn [44]. Collectively, these findings indicate that *B. velezensis* has substantial application value in improving the aquaculture environment and reducing the risk of diseases associated with ammonia nitrogen stress.

## 5. Biosafety and Ecological Risks of *B. velezensis*

Although *B. velezensis* exhibits significant potential as an antibiotic alternative in aquaculture, its large-scale application also raises biosafety concerns and potential ecological risks that warrant careful evaluation. Current safety assessments indicate that most *B. velezensis* strains demonstrate a favorable safety profile, as they typically exhibit γ-hemolysis, are sensitive to multiple antibiotics (e.g., tetracycline, erythromycin, chloramphenicol, ciprofloxacin), and lack known virulence factor genes or major enterotoxin genes [79]. Genomic analyses have further confirmed the absence of transferable antibiotic resistance genes or mobile conjugative plasmids in several *B. velezensis* strains, suggesting a low risk of horizontal gene transfer with surrounding pathogens [80]. However, strain-specific functional differences represent a critical safety consideration. Not all *B. velezensis* strains share identical biosafety characteristics; some isolates have been reported to exhibit resistance to certain antibiotics or to carry specific resistance genes [81,82]. This variability underscores the necessity of comprehensive safety evaluations for each candidate strain prior to commercial application, including antibiotic susceptibility testing, hemolytic activity assays, and genomic screening for virulence and resistance determinants [83].

## 6. General Applications of *B. velezensis* Beyond Aquaculture

*B. velezensis*, as a multifunctional probiotic, has found applications far beyond the aquaculture sector, demonstrating broad prospects in livestock and poultry farming, plant disease control, food fermentation, and industrial production. In livestock and poultry farming, *B. velezensis* can be used as a green feed additive. It produces digestive enzymes such as proteases, amylases, and cellulases to improve feed nutritional quality and enhance animals’ digestion and absorption of nutrients [84,85]. Meanwhile, its secreted antimicrobial substances effectively inhibit the growth of pathogenic bacteria such as *Escherichia coli*, *Staphylococcus aureus*, and *Clostridium perfringens*, regulate intestinal microbiota balance, and enhance animal immunity, thereby improving the growth performance of piglets, calves, lambs, and other livestock while reducing the incidence of diseases such as diarrhea [86,87,88]. In the biological control of plant diseases, *B. velezensis* produces various lipopeptide antibiotics, including iturin, fengycin, and surfactin, demonstrating significant control efficacy against multiple plant diseases such as mango anthracnose, cucumber downy mildew, peanut southern blight, wheat sharp eyespot, and rapeseed sclerotinia stem rot [89,90,91,92]. In the field of food fermentation, *B. velezensis*, owing to its salt tolerance and high-yield production of proteases and lipases, is used as a starter culture to shorten fermentation time, improve product flavor, and enhance nutritional value. It has been applied in the production of fermented foods such as soy sauce, soybean paste, and fish sauce [93]. In summary, *B. velezensis*, with its excellent biological characteristics and multifunctionality, holds significant application value in agriculture, food, environmental protection, and other fields.

## 7. Challenges and Future Directions

Despite the promising prospects of *B. velezensis*, several challenges remain, including strain-dependent functional variability, incomplete understanding of its multi-pathway in vivo mechanisms, inconsistent efficacy under complex production conditions, and the need for optimized commercial production to ensure spore yield and product stability. To address these issues, future research should focus on four priority areas: (1) elucidating host–probiotic–pathogen interactions and engineering superior strains via multi-omics and gene editing; (2) improving survival under harsh conditions (e.g., feed pelleting, gastric acid) through novel formulations such as microencapsulation; (3) exploring synergistic combinations with prebiotics, enzymes, or herbal compounds for enhanced efficacy; and (4) conducting large-scale field trials across diverse systems and conditions to establish standardized application protocols. Collectively, these efforts will help position *B. velezensis* as a viable antibiotic alternative in sustainable aquaculture.

## Figures and Tables

**Table 1:** Application effects of *Bacillus velezensis* in different aquatic animal species.

Effective Dose/Mode of Application (Strain)	Target Host	Duration	Probiotic Effects	Reference
Dietary supplementation at 4 × 10^7^ CFU/g (AP193)	Channel catfish (*Ictalurus punctatus*) fingerlings (~15–40 g)	10 weeks	In aquaria: Best weight gain (6.78 g/fish) and FCR (1.04) among tested strains; reduced *Edwardsiella ictaluri* mortality from 62.1% to 47.8%; stabilized intestinal microbiota. In ponds: Improved growth (40.4% and 32.6% increases for two fish populations); improved water quality (TP ↓19%, TN ↓43%, nitrate ↓75%).	[47]
Dietary supplementation at 1 × 10^7^ CFU/g (BV007)	Pacific white shrimp (*Litopenaeus vannamei*) ~0.32 g	8 weeks	Improved growth; increased α-amylase activity; reduced GPT and GOT. Enhanced immune responses: increased phagocytic activity, respiratory burst, SOD, CAT, and AKP activities; upregulated *cat*, *sod*, *crustin*, *penaeidin 3 a*, *lgbp*, and *lec* genes. Modulated gut microbiota: increased *Bacillus* abundance, reduced *Vibrio* and *Pseudoalteromonas*. Improved intestinal morphology. Post-*V. parahaemolyticus* challenge, cumulative mortality reduced from 97.2% (control) to 30.6% (BV2).	[48]
Immersion bath at 1 × 10^6^ CFU/mL and 1 × 10^7^ CFU/mL (CPA1–1)	Oriental river prawn (*Macrobrachium nipponense*), ~2.83 g	20 days	Increased ACP, AKP, and SOD activities; upregulated immune-related genes (*Crustin*, *Dorsal*, *Hemocyanin*, *p38*, *Prophenoloxidase*, *Stat*); degraded ammonia nitrogen and nitrite in culture water. Improved survival against non-O1 *Vibrio cholerae* from 13.3% (control) to 53.3% (1 × 10^6^ CFU/mL) and 73.3% (1 × 10^7^ CFU/mL).	[44]
Dietary supplementation at 1 × 10^7^ CFU/g (CYS06)	Grass carp (*Ctenopharyngodon idella*) ~11.8–14.8 g	4 weeks	In vitro: Broad-spectrum antagonistic activity against 19 aquatic pathogens; produced amylase, cellulase, protease, and lipase. Genome revealed 8 antimicrobial gene clusters and 156 CAZyme genes; no virulence or enterotoxin genes. In vivo: Safe for grass carp. Post-*A. hydrophila* challenge, survival increased (10^7^ group: 73.3% vs. 26.7% control).	[49]
Dietary supplementation (incorporated in feed) at 1 × 10^8^ CFU/mL (D-18)	European seabass (*Dicentrarchus labrax*) 35–200 g	20 days	In vitro: Inhibited *Vibrio anguillarum* growth by 30%; survival: 1.5 h in 10% bile and 5% at pH 4; adhered to intestinal mucus (60.3%) and reduced pathogen adhesion by 60%. Safety: No histopathological damage or bacterial recovery from organs. In vivo: Improved survival against *V. anguillarum* challenge from 35% (control) to 78%.	[40]
Dietary supplementation at 1 × 10^5^ CFU/mL (via activated powder mixed with feed) (DH82)	Pacific white shrimp ~13.3 g	28 days	In vitro: DH82 supernatant/pellets inhibited *Vibrio parahaemolyticus*; QQ enzymes (AiiA/YtnP) downregulated QS regulators (AphA, OpaR) and virulence factor (*tlh*). In vivo: enriched gut *Bacillus* (7.27% vs. 0.03%) and reduced *Vibrio* (0.38% vs. 5.57%); maintained ACP, AKP and SOD activities under challenge; alleviated intestinal and muscle damage; improved survival against co-injection.	[39]
Dietary supplementation at 1 × 10^8^ CFU/g (FiA2)	Crucian carp (*Carassius auratus*) ~12.75 g	30 days	In vitro: Produced oxydifficidin with broad-spectrum activity and high stability; MIC/MBC against *A. salmonicida* at 15%/16%; no hemolysis or cytotoxicity. Increased WGR (15.9% vs. 8.44%) and upregulated *IGF-1*/*IGF-2*. Upregulated *IL-8*, *IL-1β*, *LYZ*, *C3*, *TNF-α*, and *IgM* in head kidney, liver, and spleen. Increased Actinobacteriota and Firmicutes; decreased *Plesiomonas*. Post-*A. salmonicida* challenge survival increased from 18% to 45%.	[50]
Dietary supplementation at 1 × 10^7^ CFU/g (GY65)	Mandarin fish (*Siniperca chuatsi*), ~76.65 g	8 weeks	In vitro: Hydrolyzed various starches; antagonistic activity against many fish pathogens. Genome revealed 7 amylase genes and 12 secondary metabolite gene clusters. In vivo: Improved growth (WGR, SGR, VSI, CF); increased amylase, protease, lysozyme, SOD, CAT activities; upregulated *HSP70*, *TNF-α*, and *IL-8* in kidney and spleen. Post-*A. hydrophila* challenge survival increased from 37.8% to 62.2–64.5%.	[51]
Dietary supplementation at 1 × 10^9^ CFU/g (JW)	Crucian carp ~25 g juvenile	4 weeks	Increased serum ACP, AKP, and GSH-Px; modulated cytokine expression (*IFN-γ*, *TNF-α*, *IL-4*, *IL-10* ↑; *IL-12* ↓) in head kidney. Improved survival against *Aeromonas hydrophila* from 30% (control) to 67% and 80%. Genome revealed 4 bacteriocin, 3 PKS, and 5 NRPS gene clusters.	[41]
Dietary supplementation at 1 × 10^7^ CFU/g (K2)	Hybrid grouper (*Epinephelus lanceolatus* ♂ × *E. fuscoguttatus* ♀) ~5 g juvenile	28 days	In vitro: Broad-spectrum antimicrobial activity against fish pathogens; no hemolysis or in vivo pathogenicity. Increased serum ACP (weeks 1–2); upregulated *lysozyme*, *piscidin*, *IgM*, and *MyD88*; downregulated *IL-8*, *CC chemokine 1*, and *TLR3*; no effect on AKP, C3, TLR5, or growth. Post-*Vibrio harveyi* challenge survival increased from 25% to 55%.	[52]
Dietary supplementation at 1 × 10^9^ CFU/g(LF01)	Nile tilapia (*Oreochromis niloticus*) ~4.0 g	9 weeks	In vitro: Inhibited 9 fish pathogens; active compounds showed high thermal, pH, UV, protease stability. In vivo: Improved growth (WGR, SGR); increased serum LZY and SOD; upregulated immune genes (*lyzc*, *C3*, *MHC-IIβ*) in intestine, head kidney, and gill. Post-*Streptococcus agalactiae* survival: control 11.11% (6 wk)/20.8% (9 wk); continuous feeding 64.4% (6 wk)/79.2% (9 wk) with RPS of 60.7% and 70.6%. Reduced intestinal pathogens (*Edwardsiella, Plesiomonas*).	[43]
Dietary supplementation at 1 × 10^10^ CFU/kg feed; (LG37)	Grass carp ~20–30 g	4 weeks	Water quality: LG37 biofilm degraded ammonia nitrogen by 55.5% and feed protein by 73.6% in wastewater within one week. Growth: Utilized 10 sugars, 17 amino acids, and most organic nitrogen sources. Ammonia tolerance: Dietary LG37 increased safe ammonia (non-ionic ammonium) concentration from 3.37 to 5.11 mg/L (51.6% increase); 96 h LC_50_ increased from 33.7 to 51.1 mg/L.	[53]
Dietary supplementation at 7 × 10^7^ CFU/g (LH023)	Grass carp ~49–55 g	6 weeks	In vitro: Antagonistic activity against *Staphylococcus aureus* and *A. hydrophila*; sensitive to 12 antibiotics; no hemolysis. In vivo: Improved growth and digestive enzymes (α-amylase, lipase, trypsin, AKP); downregulated intestinal pro-inflammatory genes and upregulated anti-inflammatory, antioxidant, and intestinal barrier genes; increased beneficial gut bacteria. Post-*A. hydrophila* challenge survival reached 48% vs. 0% in control.	[54]
Dietary supplementation at 1 × 10^8^ CFU/g (R-71003)	Common carp (*Cyprinus carpio*) ~70 g	60 days	In vitro: Produced multiple digestive enzymes; antagonistic against many fish pathogens; tolerated pH 2, 0.3% bile salts, and 80 °C; high auto-aggregation and hydrophobicity. Genome revealed adhesion, amino acid/vitamin synthesis, digestive enzyme genes, and antimicrobial clusters. Safety: Sensitive to 10 antibiotics; γ-hemolysis; no in vivo pathogenicity. In vivo: Post-*A. hydrophila* challenge survival increased from 50% to 75%. Sodium gluconate (0.6–0.9%) promoted growth and upregulated *dnaA*/*yabD*.	[55]
In vitro characterization; safety evaluation via intraperitoneal injection and immersion challenge at 1 × 10^8^ and 1 × 10^9^ CFU/mL (STPB10)	Common carp	30 days	Strong biofilm formation (enhanced by glucose); γ-hemolytic; survived pH 2–4 (31–75% at 6 h), 0.3% bile (>99%), 0.5–8% NaCl, 15–35 °C. High mucus adhesion (63.7%); reduced pathogen adhesion (*A. hydrophila* 26.7%, *A. veronii* 45.9%, *A. salmonicida* 58.6%, *V. anguillarum* 57.1%). Strong antagonistic activity against *A. salmonicida* (30 mm), *A. veronii* (26 mm), *V. anguillarum* (22 mm), and *A. hydrophila* (15 mm). Sensitive to multiple antibiotics. Safety: No mortality or clinical signs in common carp at 10^8^–10^9^ CFU/mL; no bacterial recovery from organs.	[56]
Dietary supplementation at 1 × 10^8^ CFU/g (T1 and T23)	Zebrafish (*Danio rerio*) ~38 mg	4 weeks	T23 promoted weight gain, lipase activity, intestinal morphology (villus length, muscle thickness); reduced serum LPS and TNF-α; upregulated hepcidin. T1 showed stronger protease but no growth promotion; downregulated *TNF-α*, *IL-1β*; upregulated *hepcidin*. Both improved survival against *Aeromonas veronii*, with T23 better. Both altered gut microbiota, affecting growth/immunity genes in GF-zebrafish. Direct immersion: T1 upregulated *TNF-α*; T23 affected *IL-1β* and *IL-10*.	[57]
Dietary supplementation with solid fermentation product at 1 × 10^6^ CFU/g (T23)	Zebrafish ~60 mg	6 weeks	Enhanced non-specific immunity (T-AOC, lysozyme, C3, C4); alleviated HFD-induced hepatic steatosis and inflammation by downregulating lipogenesis genes (*PPARγ*, *Srebp-1c*, *Fas*) and pro-inflammatory cytokines (*TNF-α*, *IL-6*, *IL-1β*); reduced intestinal injury. Gut microbiota: increased *Cetobacterium* abundance.	[58]
Dietary supplementation with fermentation product at 1%, 3%, and 5% (*v*/*w*) (optimal: 1% for growth, 3% for disease control) (V4)	Rainbow trout (*Oncorhynchus mykiss*) ~200 g	2 months	In vitro: Inhibited *A. salmonicida* and *A. hydrophila*; active compounds (iturin A, macrolactin A, oxydifficidin) caused cell membrane disruption and lysis; genome revealed gene clusters for iturin, macrolactin, and difficidin (~8.4% of genome). In vivo: 1% addition reduced FCR by 19.5%, increased WGR by 71.2% and SGR by 58.6%, reduced mortality by 63.6%; 3% addition reduced mortality by 81.9%. Compounds showed high thermal/pH/enzyme stability but UV sensitivity.	[59]
Dietary supplementation: *B. velezensis* V4 5 × 10^6^ + *Rhodotorula mucilaginosa* 5 × 10^7^ CFU/g	Atlantic salmon (*Salmo salar*) ~180 g juvenile	62 days	Improved FBW, WGR, FCR, and SGR; reduced mortality (from 8.57% to 2.86–4.76%). Elevated t-SOD, GSH, GSH-Px, CAT, T-AOC, ACP, and IgM; decreased NO, GPT, GOT, and MDA. Post-*Aeromonas salmonicida* challenge, T1 group showed 73.3% survival vs. 6.7% in control; improved t-SOD, CAT, ACP, lysozyme, IgM, and cortisol levels.	[60]
Dietary supplementation at 1 × 10^8^ CFU/g (WLYS23)	Hybrid snakehead (*Channa maculata* ♀ × *C. argus* ♂) ~40–43 g	4 weeks	In vitro: Broad-spectrum antagonistic activity against 20 fish pathogens; produced amylase, cellulase, protease, lipase. Genome revealed 7 antimicrobial gene clusters; no virulence genes. In vivo: cell-free extract feeding improved *Aeromonas schuberti* survival from 13.3% to 63.3%; strain feeding improved survival from 23.3% to 60%. Safe for snakehead and zebrafish.	[19]
Dietary supplementation at 1 × 10^7^ CFU/g	Rainbow trout ~19.4 g	30 days	Improved growth and intestinal morphology; enhanced serum immune enzymes (ACP, AKP, lysozyme, SOD, CAT, POD). Upregulated immune and growth genes; enriched ribosome, oxidative phosphorylation, and phagosome pathways. Increased beneficial gut bacteria and metabolites linked to starch/sucrose metabolism and TCA cycle. Post-*A. salmonicida* challenge survival significantly improved.	[61]
Dietary supplementation at 1 × 10^9^ CFU/g	Pacific white shrimp ~3 g	60 days	Improved growth, survival, and body composition. Increased digestive enzymes (lipase, amylase, protease), antioxidant enzymes (SOD, CAT), immune parameters (IgM, BA%, AKP, ACP); decreased MDA, triglycerides, cholesterol, HDL, LDL, AST, and ALT. Upregulated *SOD* (G3), lysozyme, and serine proteinase genes. Hepatopancreas showed increased B and R cells.	[45]

Table footnotes: ACP, acid phosphatase; AKP, alkaline phosphatase; ALT, alanine aminotransferase; AST, aspartate aminotransferase; BA%, bacteriolytic activity percentage; CAT, catalase; CAZyme, carbohydrate-active enzyme; CF, condition factor; FCR, feed conversion ratio; FBW, final body weight; GF, germ-free; GOT, glutamic-oxaloacetic transaminase; GPT, glutamic-pyruvic transaminase; GSH, glutathione; GSH-Px, glutathione peroxidase; HDL, high-density lipoprotein; HFD, high-fat diet; LC_50_, median lethal concentration; LDL, low-density lipoprotein; LPS, lipopolysaccharide; LZY, lysozyme; MBC, minimum bactericidal concentration; MDA, malondialdehyde; MIC, minimum inhibitory concentration; NO, nitric oxide; NRPS, non-ribosomal peptide synthetase; PKS, polyketide synthase; POD, peroxidase; QQ, quorum quenching; QS, quorum sensing; RPS, relative percent survival; SGR, specific growth rate; SOD, superoxide dismutase; T-AOC, total antioxidant capacity; TCA, tricarboxylic acid; TN, total nitrogen; TP, total phosphorus; t-SOD, total superoxide dismutase; VSI, viscerosomatic index; WGR, weight gain rate. Arrows (↓) indicate a decrease in the respective parameter.

## Data Availability

No new data were created or analyzed in this study. Data sharing is not applicable to this article.
